# Correction: Adaptive political surveys and GPT-4: Tackling the cold start problem with simulated user interactions

**DOI:** 10.1371/journal.pone.0336799

**Published:** 2025-11-13

**Authors:** The PLOS One Staff

The figures in the S1 Text are missing. Please view the correct S1 Text below. The publisher apologizes for the error.

## Supporting information

S1 Text Additional Figures and Tables. (PDF)
