# Supplementary material for: Correction: Adaptive political surveys and GPT-4: Tackling the cold start problem with simulated user interactions
Source: PLoS One. 2025 Nov 13;20(11):e0336799. doi: 10.1371/journal.pone.0336799 (PMC12614500; doi:10.1371/journal.pone.0336799)
Supplement: S1 Text — (PDF) [file pone.0336799.s001.pdf]

Additional Tables and Figures

Table 3. Party abbreviations, translations and political orientations. The table shows the eight major parties in the canton of Zurich, Switzerland, ordered by political position.

Abbreviation	Full German Name	Official English Translation	Political Position
SP	Sozialdemokratische Partei der Schweiz	Social Democratic Party	Left
Greens	Grüne Partei der Schweiz	Green Party	Left
GLP	Grünliberale Partei der Schweiz	Green Liberal Party	Left-Liberal
EVP	Evangelische Volkspartei der Schweiz	Evangelical People's Party of Switzerland	Centrist
Centre	Die Mitte	The Centre	Centrist
FDP	Freisinnig-Demokratische Partei	The Liberals	Liberal-Right
EDU	Eidgenössisch-Demokratische Union	Federal Democratic Union	Right-Conservative
SVP	Schweizerische Volkspartei	Swiss People's Party	Right

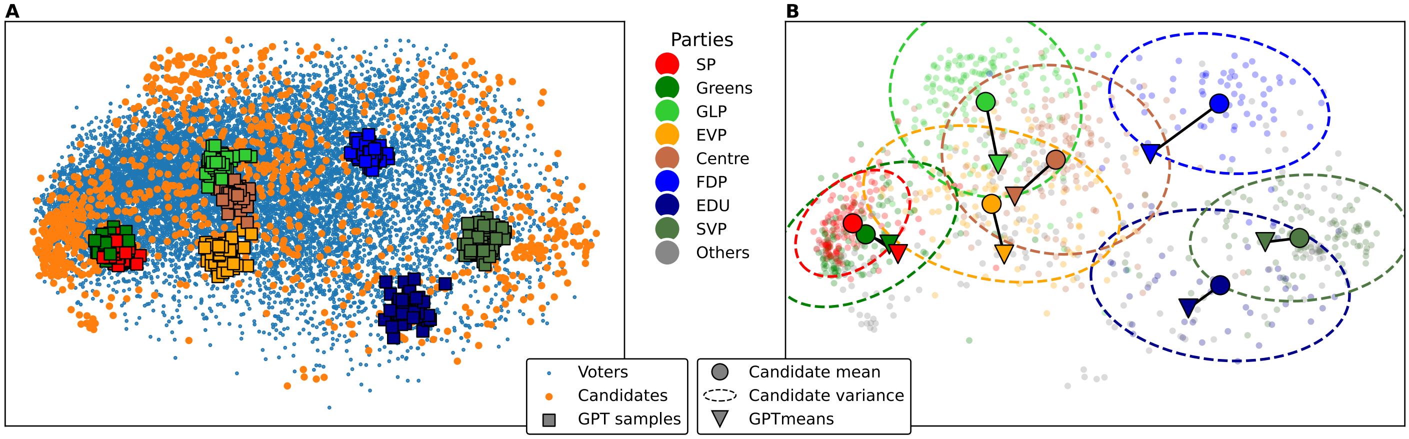

Fig 8. Data generation results with GPT-4. A: The PCA projection of the candidates (orange) shows the distribution of candidates in a two-dimensional space. In blue, the voters' dataset projected onto the same principal components shows a more unimodal distribution: it has the highest density in the centre and evenly fades out to all directions. The coloured clusters correspond to the GPT-4 generated answers with their respective party membership. B: In the same two-dimensional space, the candidates are coloured by their party membership. For each party, a normal distribution is fitted. The dashed ellipses correspond to their 95%-confidence interval, while the coloured circles correspond to the party-mean. The triangles represent the means of the GPT-4 samples.

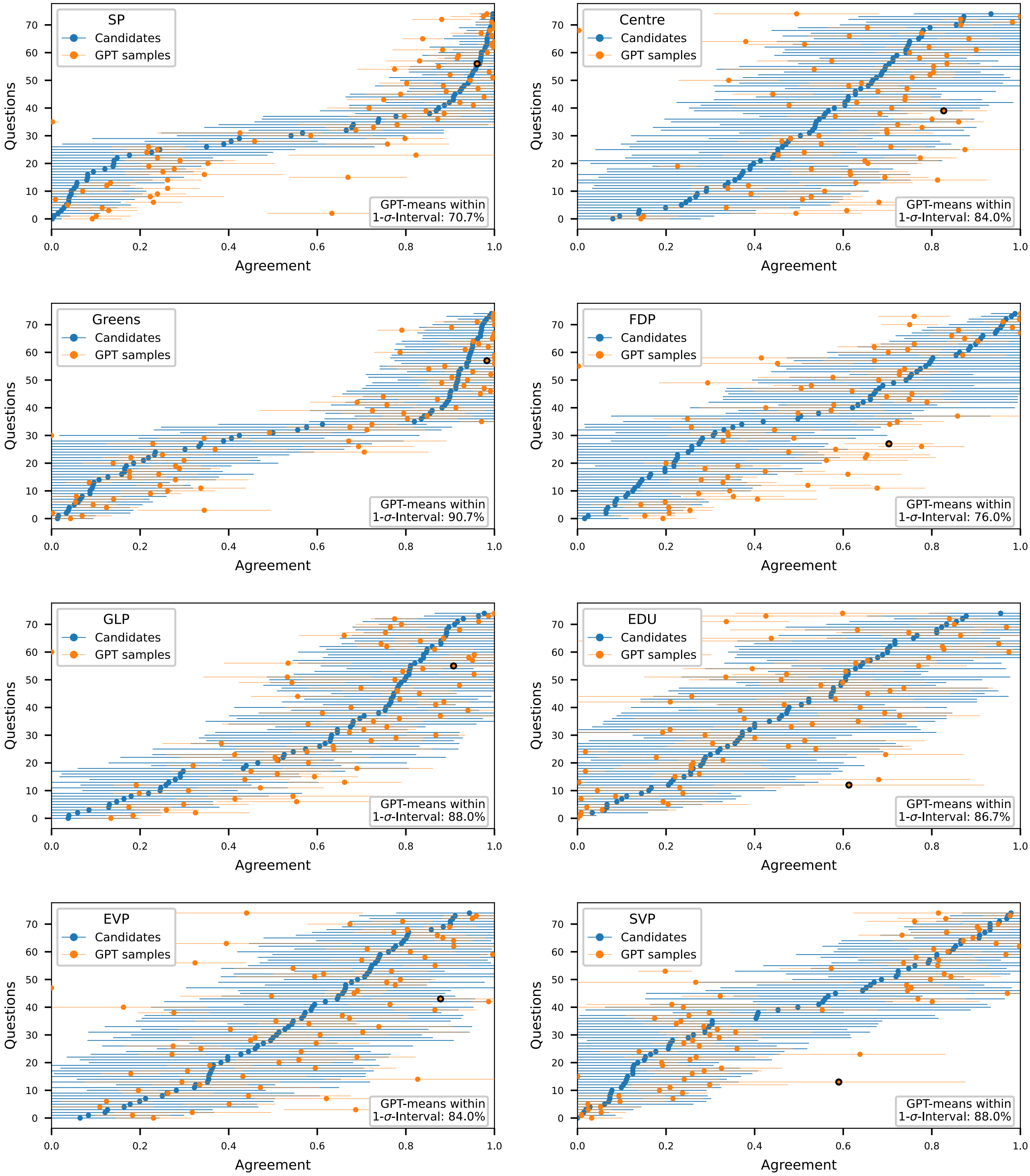

Fig 9. GPT-4 samples compared to candidates' responses. For each question, the mean and standard deviation of the candidates of the respective party are shown by the blue dots and horizontal error bars. In orange, the GPT-4 means and standard deviations for that question and party are shown. The question "Should direct payments only be granted to farmers with proof of ecological performance?" is highlighted by the black circle.

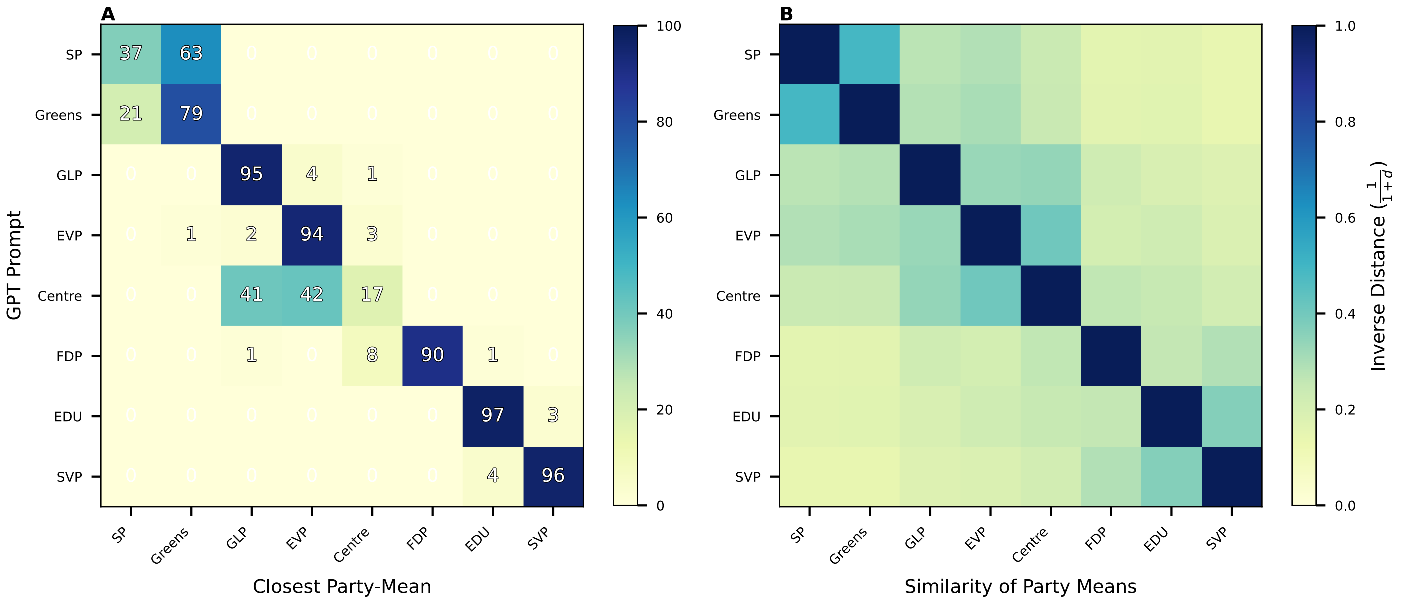

Fig 10. Similarity of GPT samples and their respective party-mean. A: Each cell in the heatmap shows the percentage of GPT samples per party closest to the respective party mean on the x-axis. For example, 63% of the GPT samples prompted to be a member of SP are closest to the party-mean of the Greens. In contrast, 96% of the GPT samples prompted to be a member of SVP are closest to the party-mean of the SVP. B: The pairwise inverse distance of the party-means shows the similarity of different parties.

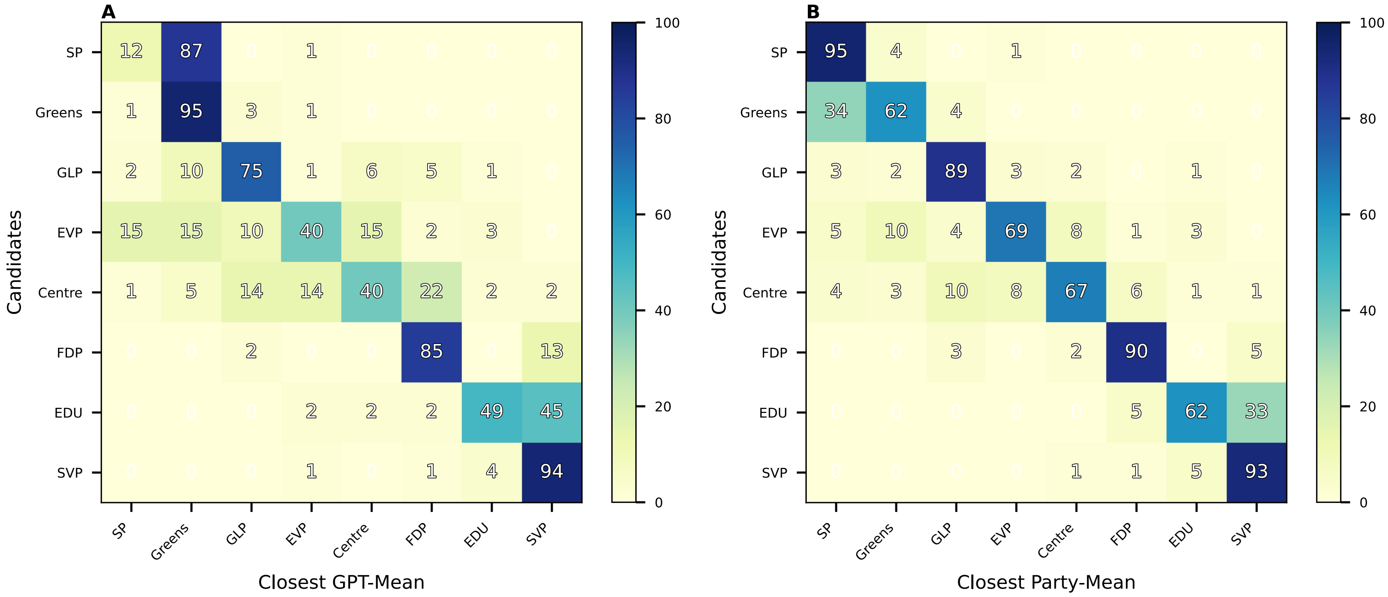

Fig 11. Similarities of the individual candidates. A: The heatmap shows the percentage of candidates closest to the respective GPT-mean on the x-axis. For example, 87% of the SP candidates are closest to the GPT-mean of the Greens. In contrast, 86% of the FDP candidates are closest to the GPT-mean prompted to belong to the FDP. B: The heatmap shows the percentage of candidates closest to the respective party-mean on the x-axis. For example, 33% of the EDU candidates are closest to the party-mean of the SVP. In contrast, 89% of the GLP candidates are closest to their own party-mean.

Table 4. GPT-4 results for different temperature (noise) parameters. For each temperature, the mean and standard deviation of the respective GPT samples are computed. The mean is used to measure the distance to the respective party-mean (Mean Distance). The standard deviation is used to quantify the variance of the samples across multiple trials for the same party (Response Variance). For all GPT-4 responses that could not be transferred to an integer between 0 and 100, e.g., when GPT-4 elaborated on its answer or avoided a rating, the value was considered missing (Missing Values).

Temperature	Mean Distance	Response Variance	Missing Values
1.00	0.160	0.076	0 (0.00%)
1.25	0.163	0.088	1 (0.02%)
1.50	0.165	0.100	0 (0.00%)
1.75	0.170	0.108	13 (0.22%)
2.00	0.167	0.116	95 (1.58%)
Mean	0.165	0.098	21.8 (0.36%)

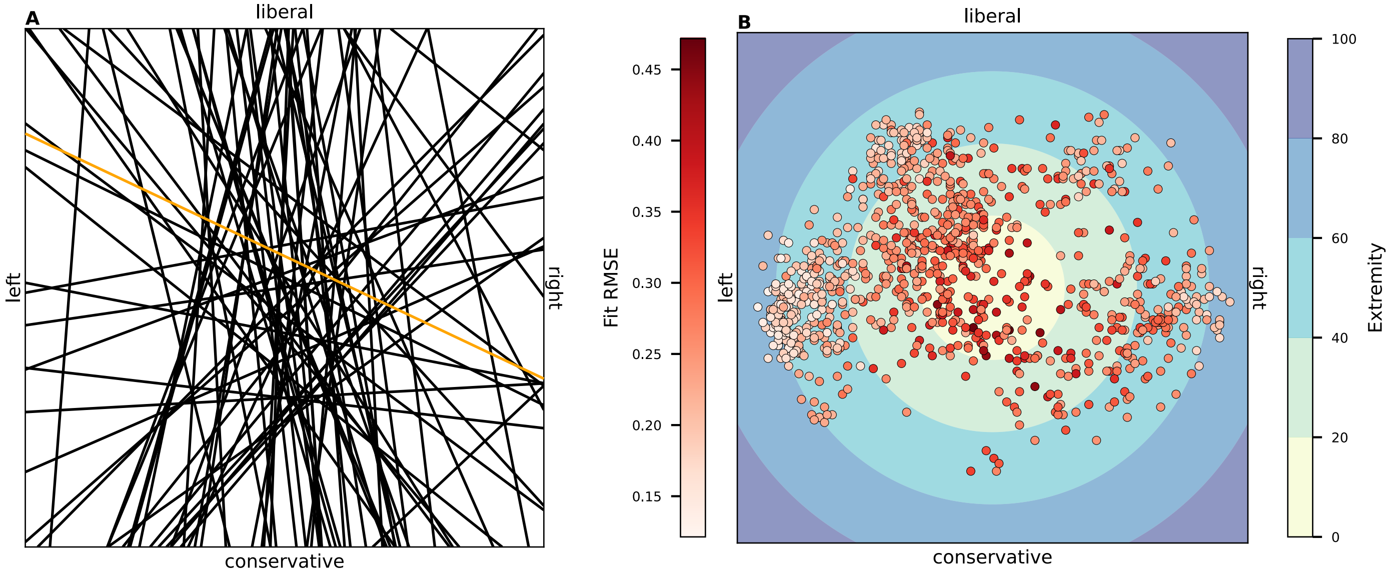

Fig 12. Model fit of the candidates' dataset A: For each question, the decision boundary of the logistic regression is shown. In orange, the question "Do you support the increase of the retirement age (e.g., to 67)?" is highlighted. B: The candidates are coloured by the fit RMSE of their embedding in the latent space of the statistical model. A higher RMSE indicates worse predictive accuracy. The background shows the extremity of the candidates computed by the distance to the origin.

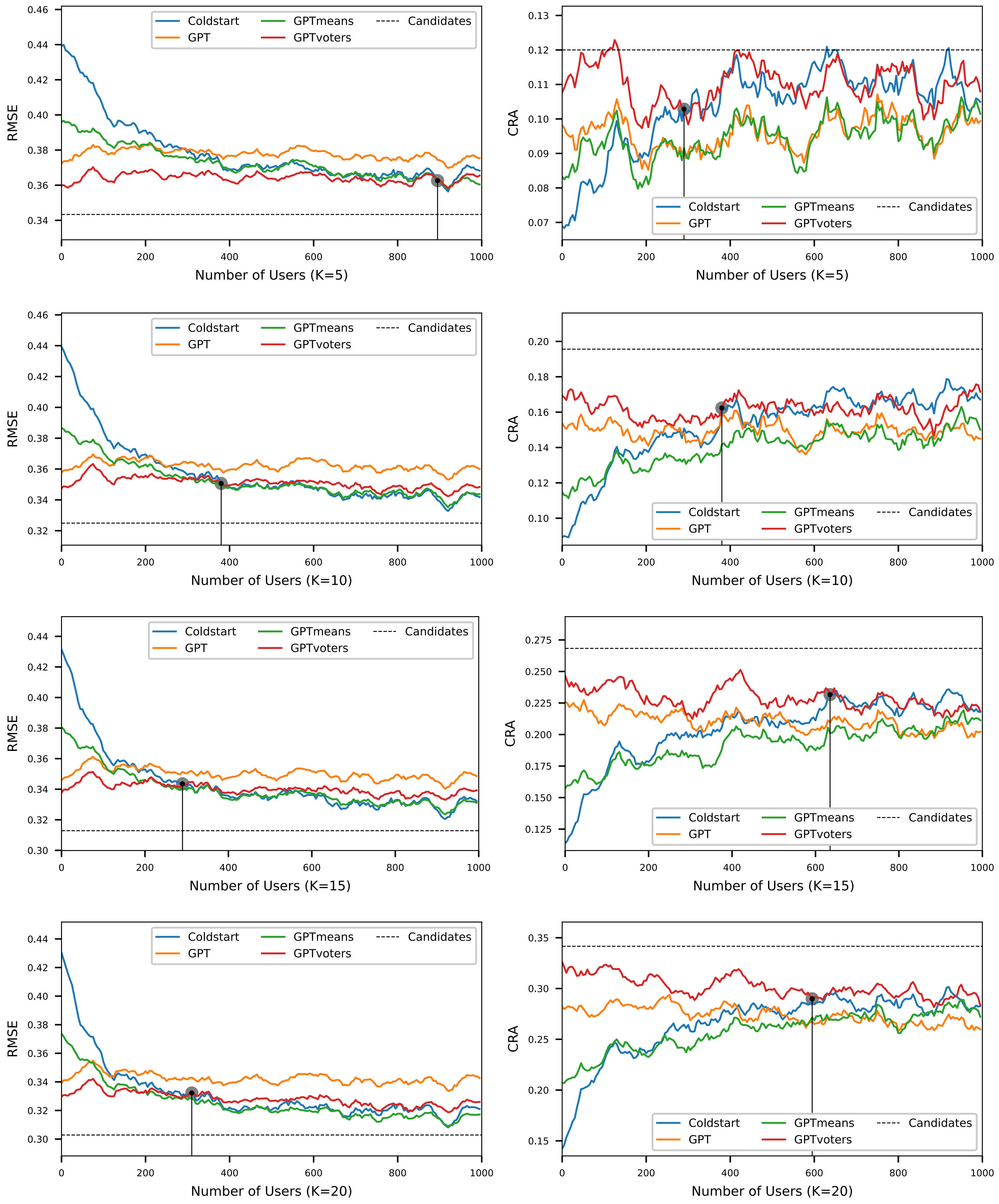

Fig 13. Results of the questionnaire simulation with different training data and values of $K \in \{5, 10, 15, 20\}$.

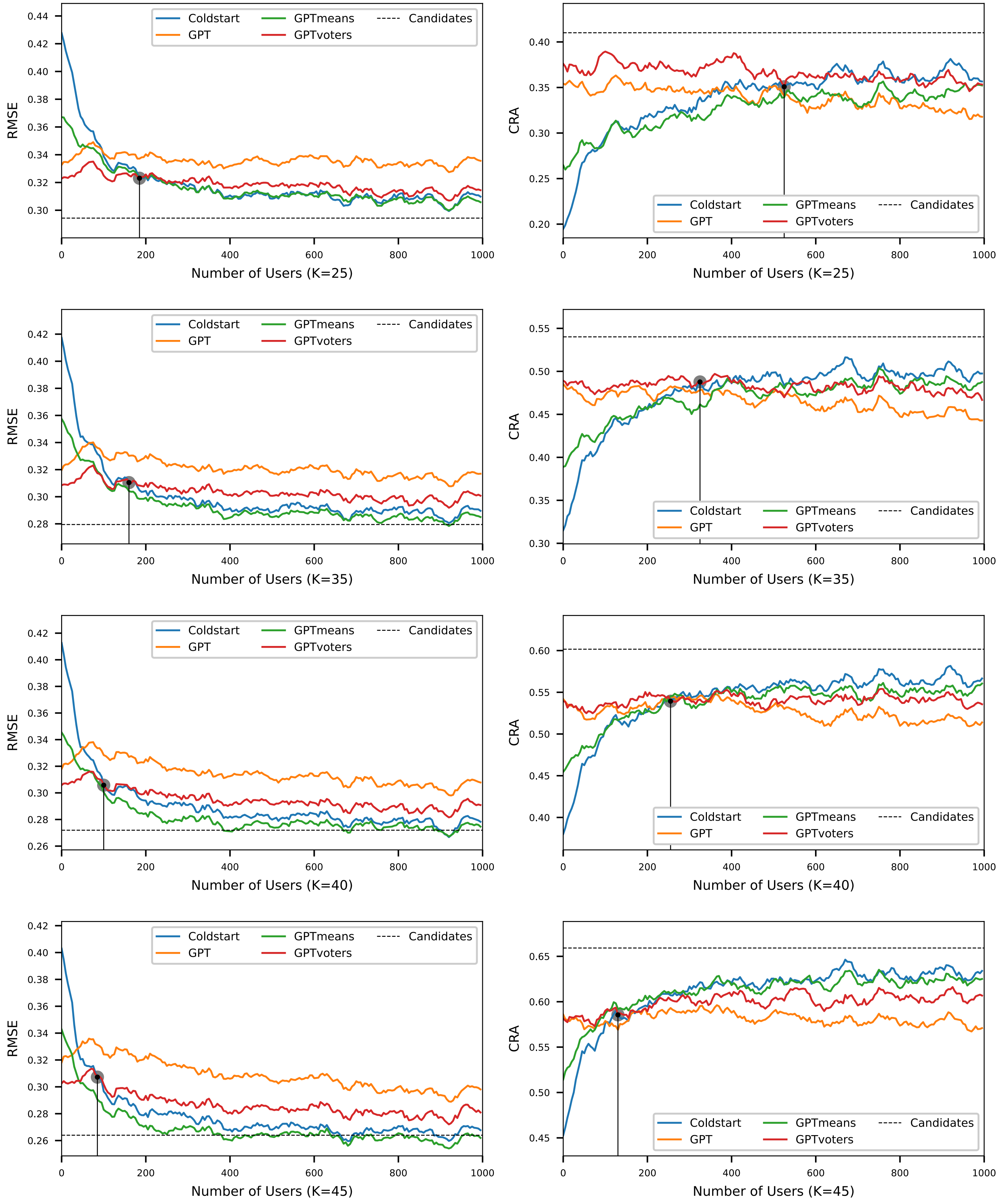

Fig 14. Results of the questionnaire simulation with different training data and values of $K \in \{25, 35, 40, 45\}$.

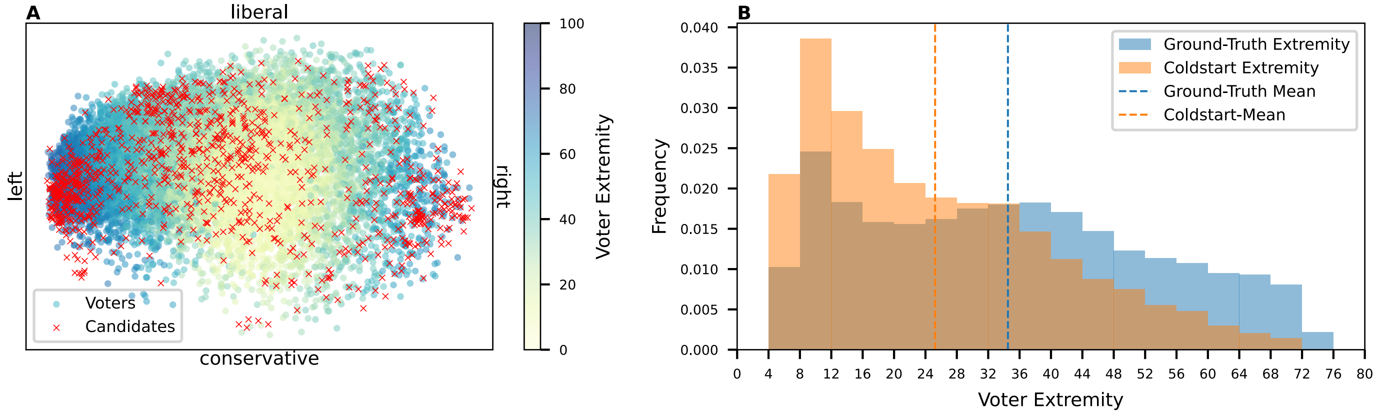

Fig 15. Extremity Estimation. A: The PCA projection of the voters' dataset is coloured by the average extremity of their recommended candidates. The candidates are shown as red crosses. B: The histogram shows the distribution of extremity across voters. In blue, the ground truth extremity is evenly distributed across the space. In orange, the estimated extremity is given by the average extremity of the recommended candidates after answering $K = 30$ questions in the adaptive questionnaire with random initialisation. In comparison, this estimation is biased towards smaller values.

Table 5. Extremity bias for different training data and numbers of questions per user. For each voter, the recommended candidates' extremity is averaged. The table shows the difference between the average extremity of recommendations with full information compared to the average extremity of recommendations after answering only K number of queries. Each column shows the result for a different training dataset.

K	Coldstart	GPT	GPTmeans	GPTvoters	Candidates
5	20.7 ± 0.3	24.9 ± 0.3	24.5 ± 0.3	22.7 ± 0.3	22.7 ± 0.3
10	17.9 ± 0.3	19.6 ± 0.3	21.6 ± 0.3	18.7 ± 0.3	16.4 ± 0.3
15	15.9 ± 0.3	15.5 ± 0.2	18.7 ± 0.3	14.4 ± 0.3	12.6 ± 0.2
20	13.4 ± 0.2	12.1 ± 0.2	15.8 ± 0.2	10.9 ± 0.2	10.0 ± 0.2
25	10.6 ± 0.2	9.4 ± 0.2	13.1 ± 0.2	8.4 ± 0.2	8.1 ± 0.2
30	9.1 ± 0.2	7.4 ± 0.2	10.9 ± 0.2	6.6 ± 0.2	6.8 ± 0.2
35	7.5 ± 0.2	5.8 ± 0.1	8.7 ± 0.2	5.3 ± 0.2	5.2 ± 0.1
40	5.9 ± 0.1	4.3 ± 0.1	7.0 ± 0.1	4.3 ± 0.1	4.0 ± 0.1
45	4.6 ± 0.1	2.9 ± 0.1	5.2 ± 0.1	3.3 ± 0.1	2.9 ± 0.1
